# The Impacts of Housing Characteristics and Built-Environment Features on Mental Health

**DOI:** 10.3390/ijerph19095143

**Published:** 2022-04-23

**Authors:** Zihan Kan, Mei-Po Kwan, Mee Kam Ng, Hendrik Tieben

**Affiliations:** 1 Institute of Space and Earth Information Science, The Chinese University of Hong Kong, Shatin, Hong Kong, China; zihankan@cuhk.edu.hk; 2 Department of Geography and Resource Management, The Chinese University of Hong Kong, Shatin, Hong Kong, China; meekng@cuhk.edu.hk; 3 School of Architecture, The Chinese University of Hong Kong, Shatin, Hong Kong, China; hktieben@cuhk.edu.hk

**Keywords:** housing characteristics, built environment, mental health, structural equation modeling

## Abstract

In this study, we examined the relationships between housing characteristics, neighborhood built-environment features, and people’s mental health in Hong Kong, an Asian city well known for its high-density and high-rise housing. The potential mediating effects of people’s perceived living environment were also considered in the analysis. We collected data from 221 participants from two communities in Hong Kong, i.e., Sham Shui Po (SSP) and Tin Shui Wai (TSW), using a stratified random sampling approach. Big datasets were also used to derive relevant built-environment features at the street block level. We used structural equation modeling to explore the complex relationships among housing characteristics, built-environment features, and mental health. The results indicate that the associations between built-environment quality and people’s mental health are weak. For communities with relatively poor housing conditions (i.e., SSP in this study), the impact of housing characteristics on mental health may be more direct; for communities with relatively good housing conditions (i.e., TSW in this study), the effect of housing characteristics on mental health may be indirect. Our findings shed light on the importance of considering different contexts in developing policies related to housing and built environment and mental health.

## 1. Introduction

The notion that features of the living environment may be related to psychological stress and mental health has a long history. In the past few decades, there has been increasing interest in the epidemiology and public health literature about how residential environments may have influences on a variety of health outcomes, and evidence has shown that the living environment has a great impact on people’s mental health [1]. On the one hand, neighborhood attributes such as inadequate housing, crowding, social disorder, violence, and lack of green spaces may function as stressors on people’s mental health [2,3,4]. On the other hand, neighborhood characteristics may also affect social connections and social support available to residents, which may further affect people’s well-being and vulnerability to or tolerance of psychological stress [1].

The impact of the living environment on mental health can be exerted on different spatial scales. At the micro-scale, housing is the fundamental component of the living environment [2]. Poor housing conditions and inadequate housing are likely to create multiple health risks that are responsible for considerable disease and deaths. Each year, millions of deaths globally are attributable to disadvantaged housing conditions [5]. Housing characteristics, including housing tenure, physical housing conditions, and living space per person, may act as sources of stressors that further influence people’s mental health and well-being [4,6]. Under crowded living conditions, people may have excessive, unwanted social interactions and perceptions of insufficient privacy, which may adversely influence their mental health [7]. Previous research on the impact of housing conditions on mental health, primarily in a Western context, has examined the quality of housing units on mental health [4,6,8]. For instance, living in residential units characterized by poor housing facilities such as non-functioning kitchen facilities, heater breakdowns in winter, and water leakage was found to be associated with a greater likelihood of depression in the U.S. [3,4]. Small living space, low housing affordability, and a lack of housing tenure were also reported to be linked with a higher risk of mental disorders in Australia, the United Kingdom, and South Korea [6,9,10]. Housing instability and disorganization were also found to be associated with positive screening for depression and generalized anxiety among women in the U.S. [11].

Past studies also showed significant associations between adverse built environments and mental health. Such macro-scale living environments influence people’s lifestyle and health behaviors including diet, physical activity and active travel, and configure the social environment, which further influences social interactions and support and thus mental health [12]. Some studies found that high-rise dwellings might affect residents’ social relationships and lead to social isolation and experience of loneliness, which may further weaken people’s mental health [13,14]. It was found that the prevalence of any adverse mental health symptoms such as depression and anxiety was higher among urban residents than among rural residents [15,16,17,18], and higher levels and speed of urbanization were significantly related to mental disorders [19,20]. It was reported in a recent study that people who live in the largest and densest cities are the least happy compared to people living in small towns and rural areas [21]. Specifically, adverse housing and neighborhood environment indicators were related to an increased risk of depressive mood in the older adults in Brazil [22]. Higher levels of green space in residential neighborhoods were found to be associated with significantly lower levels of anxiety, stress, and depression and greater mental wellbeing in U.S., Australian, and European contexts [23,24,25].

Despite the theoretical rationale for the effect of neighborhood characteristics on mental health, the results of the research regarding the relationship between neighborhood environment and health are not always consistent. For instance, it was reported in studies that higher population density was significantly correlated with depression in Augsburg, Germany and Lahore, Pakistan [26,27], while higher density was found to be negatively related to depressive symptoms for residents in Miami, U.S [28]. The inconsistent findings might be because the housing and population densities overall in many U.S. cities are moderate; higher densities may increase the ease with which people can stroll or walk in neighborhoods, thereby facilitating greater non-motorized travel [28]. It was further suggested in a study conducted in an urban area of Italy that neighborhood built-environment characteristics had a stronger effect on mental health for people who spend more time in the neighborhood [29]. The variation in the relationships between environmental determinants and health outcomes as a function of geographic location is conceptualized as the notion of spatial nonstationarity [30]. When there are nonstationary health–environment relationships, research findings at one geographic location cannot be directly generalized to other locations. As a result, it is often impossible to summarize the complex nonstationary relationships observed globally [30].

The pathways from the living environment to individual mental health are very complex and may be indirect. The effect may be mediated by the sense of place and community, individuals’ health conditions and subjective perception of the environment [31] because the public’s perception of environmental risks such as air and noise pollution and nighttime lighting is usually based on subjective assessment, which influences people’s psychological security. People with lower psychological security might experience negative emotions and mental disorders [32]. As a result, the public’s negative perception of environmental quality may induce a negative mentality when they are in the environment [33]. In the literature, it was found that annoyance caused by the environment such as noise and air pollution was associated with lower sleep quality and mental disorders for residents [34,35,36,37]. Perceptions of green spaces were also found to be a major contributor to people’s usage of green spaces and their wellbeing and mental health [38,39]. Much research has shown that residents’ perceptions of environmental risks are not always in line with the real ones [40]. It is thus suggested that evaluating both the objective and perceived built environment may be necessary when examining the relationship between the built environment and health behaviors [41].

Existing studies indicate that geographic context may have a great impact on people’s perceived living environment and mental health, and it is thus suggested that strategies to prevent mental disorders should consider the residential context [1]. However, little is known about the relationship between housing and the built environment and people’s mental health in a high-rise and high-density Asian city, and whether the relationship is the same in different geographic contexts. To fill this research gap, this study examines the association between individual housing characteristics, neighborhood built environment features, and mental health in two communities in Hong Kong, one located in an inner-city old district (i.e., Sham Shui Po), and the other a new town located in a suburban area in Hong Kong (i.e., Tin Shui Wai). The potential mediating effect of the perceived living environment is also considered in this research. In this study, we collected data from 221 subjects from the two communities using questionnaire surveys based on a stratified random sampling approach. Large-scale big datasets of the built environment were also used to derive the built-environment characteristics at the street-block level. Structural equation modeling was conducted to explore the complex relationships among residential environment features, perceived living environment, and mental health.

## 2. Method

### 2.1. Conceptual Framework and Hypothesis

**Figure 1:**
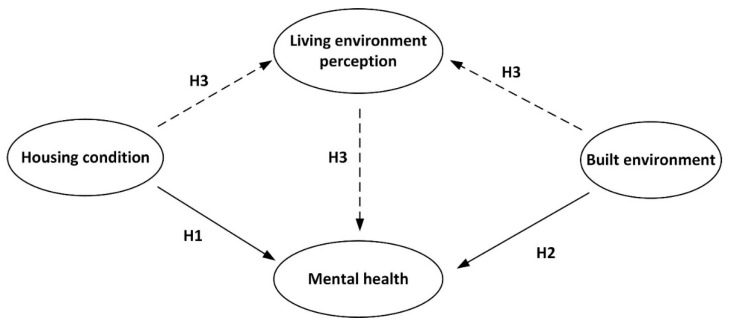
Conceptual framework and hypothesis.

The conceptual framework of this study focuses on the relationships among housing characteristics, built-environment features, perceived living environment, and mental health, as shown in Figure 1. We hypothesize that an individual’s mental health is influenced by both housing characteristics and built-environment features with the perceived living environment as a mediator, and the pathways affecting individual mental health are different in different communities. First, in line with research on the relationship between housing characteristics and mental health [4,9,10], we hypothesize that good housing condition has a positive direct effect on mental health (H1). Second, in line with previous studies on the relationship between neighborhood built environment and mental health [24,29], we hypothesize that high-quality built-environment features have a positive direct effect on mental health (H2). Third, based on previous studies on perceived neighborhood environment and mental health/psychological stress [32,34,36], we hypothesize that perceived neighborhood environment may play a mediating role in the effect of housing characteristics and built-environment features on mental health (H3).

### 2.2. Study Area and Data Sources

(1) Study area

**Figure 2:**
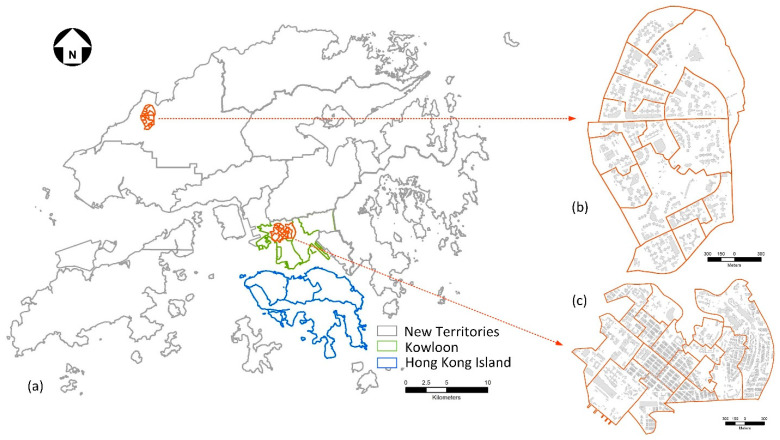
Study area: (**a**) territory of Hong Kong, (**b**) Tin Shui Wai (TSW), and (**c**) Sham Shui Po (SSP).

The study area for this research is Hong Kong, which is a metropolitan city with a very high population and housing density. Hong Kong consists of 18 districts and three regions including Hong Kong Island, Kowloon, and the New Territories (Figure 2a). As of 2021, about 7.4 million residents lived in its 1105 km^2^ territory. This study focuses on two selected communities, i.e., Sham Shui Po (SSP) and Tin Shui Wai (TSW), which are located in in the northwestern part of Kowloon and north-western area of the New Territories, as Figure 2b,c show.

Both SSP and TSW communities are median–low income areas with a mixed population composition and housing types, with a higher proportion of public rental housing in TSW and a higher proportion of private permanent housing (mostly tenement buildings) in SSP (Table 1). According to the Hong Kong Census conducted in 2016, the median monthly household income for SSP and TSW was HKD 20,000 and HKD 25,000 (the median monthly household income of all Hong Kong was HKD 25,000 in 2016).

In addition, the different characteristics of SSP and TSW in some aspects allow us to compare the results concerning the two communities. First, SSP is among the earliest developed urban areas in Hong Kong. It has severe urban decay problems with aging buildings. Some urban renewal projects are being implemented to improve the living conditions and the quality of the urban environment in SSP [42,43]. In contrast, TSW is a new town developed in the 1980s with many immigrants. Second, SSP is located in a more central area of Hong Kong with a high population density. In comparison, TSW is one of the most remote areas and is located in the northwest of Hong Kong, with a relatively lower population density.

(2) Data

With a stratified random sampling strategy, we recruited a total of 221 participants aged from 18 to 64 years to participate in the study, including 109 residents from SSP and 112 residents in TSW. The age distribution of the participants in the two communities is shown in Table 2. Overall, the average age of participants in SSP and TSW was between 35 and 38 years. It can be seen that the average age of participants in SSP is higher than that of the TSW. Especially, the males recruited in SSP were, on average, about 7 years older than the males recruited in the TSW. Table 2 also shows that female participants are older than male participants. In general, the female participants were about 5 years older than the male participants. In TSW, the female participants were, on average, 9 years older than the male participants.

The survey was conducted from March 2021 to September 2021. Each participant completed a questionnaire (as part of a larger project) to provide their demographic and socioeconomic information, as well as their housing condition, perceived living environment, and reported mental health. The whole samples of the SSP and TSW instead of strata were used to perform analysis in this study.

Built-environment features were not collected from participants; instead, they were derived from various data sources, including a 3D spatial dataset with building geometry provided by the Hong Kong Planning Department, a raster dataset of sky view calculated from multiple data sources including airborne LiDAR data, building GIS data, and land cover data, SPOT-7 Satellite images in 2017 with a spatial resolution of 6 m, and land-use dataset with 27 land-use types and with a spatial resolution of 10 m × 10 m from the Hong Kong Planning Department. The built-environment features were aggregated at the Large Street Block Group (LSBG) level, which is a spatial unit delineated by the Hong Kong Planning Department and used by the census. An LSBG is a group of street blocks with similar demographic and built-environment characteristics.

### 2.3. Measures and Statistical Model

#### 2.3.1. Measures

The selection of indicators of the housing characteristics and built-environment features used in this analysis was guided by previous research and theory linking housing and neighborhood stressors to poor mental health.

(1) Housing Characteristics

Housing is a fundamental social determinant of health, and the link between housing and health is widely acknowledged in the literature [2,4,6,9,10]. Three main characteristics of housing have been explored, namely dwelling conditions, housing affordability, and housing tenure [10]. In line with the literature, housing characteristics were assessed in this study by variables including housing tenure, type of housing, housing affordability stress, and living space per person. For each measure of housing characteristics, participants’ answers and coded values are shown in Table 3.

First, the literature indicates that housing tenure is an important factor affecting people’s mental health. Homeownership derived from higher socioeconomic status may give residents a greater sense of comfort and thus reduce their economic and psychological stress [6,9,10]. In this study, housing tenure was assessed by participants’ responses to the question “Is your home rented or owned.” In Table 3, a higher coded value of the answer indicates a better housing tenure condition.

Second, there is strong empirical evidence about associations between poor housing affordability and poor mental health, and the association may be different between homeowners and private renters, with private renters in unaffordable housing experiencing poorer mental health [44,45]. As a result, this study assessed participants’ housing affordability stress by a continuous variable derived as a ratio obtained by dividing the answer to the question “How much is the monthly mortgage or rent for your residential unit” by the answer to the question “Your monthly household income”.

Third, dwelling conditions such as poor housing facilities and overcrowding are a major risk factor for family disruption and adverse mental health symptoms [8]. This study characterized participants’ dwelling conditions by collecting their types of housing and living area per person in a household. Type of housing was assessed by participants’ responses to the question “What is your housing type.” In Table 3, the higher the coded value of the answer, the better the housing tenure condition and the facilities and living conditions the house type has [46]. In addition, living area per person was used to characterize the crowdedness of a housing unit, which is a continuous variable derived by dividing the answer to the question “What is the size of your residential unit” by the answer to the question “How many people living in your residential unit”.

The Cronbach alpha for the four questions is 0.60, indicating acceptable internal reliability [47,48]. The Comparative Fit Index (CFI) of these four variables of housing characteristics assessed by the Confirmatory Factory Analysis (CFA) is 0.94 (>0.90), indicating that the fitting result between the four observed variables and housing characteristics is good, and the latent variable of housing characteristics is set up reasonably.

(2) Built-Environment Features

Built-environment features are associated with people’s mental health by influencing people’s lifestyles, health behaviors, and social interactions [41,49]. In this study, built-environment features were assessed based on the variables of building density, greenspaces, sky view, and land-use mix at the LSBG level.

First, building density determines how crowded a neighborhood is, and research has identified it as a feature of the external living environment that affects mental health [7]. In this study, building density was derived from the 3D spatial dataset with building geometry and height provided by the Hong Kong Planning Department.

Second, the sky view factor was assessed to partially reflect the quality of the neighborhood environment [50], which is the ratio of the area of sky visible from a location on the ground to the sky area that is potentially available to the location. A higher value of the sky view factor at a location is associated with higher exposure to natural light, which has a positive effect on people’s physical and mental health. More sky view may also increase the cooling capacity of the neighborhood environment and alleviate the effects of urban heat island, which was found to be a contributing factor to suicide mortality in the high-density city of Hong Kong [50]. In this study, the sky view factor was derived based on a 10 m × 10 m raster dataset calculated from multiple data sources including airborne LiDAR data, building GIS data, and land cover data in a previous study [51].

Third, the relationships between more green spaces and lower levels of anxiety, stress and depression in the literature are well established [23,24,25]. In this study, the greenspace indicator was calculated using the sum of the Normalized Difference Vegetation Index (NDVI) derived from SPOT-7 Satellite images in 2017 with a spatial resolution of 6 m.

Lastly, land-use diversity was reported to have positive effects on both mental health and subjective wellbeing in a recent study conducted in Hong Kong, with protective effects on people’s mental health through easy access to resources to meet multiple needs [52]. In this study, data of a variety of land-use types were acquired from a raster land-use dataset with a spatial resolution of 10 m × 10 m from the Hong Kong Planning Department. The land-use mix index (*LUMI*) was calculated as the degree of the land-use mix for each LSBG based on the notion of entropy, as Equation (1) shows.

(1) $$LUMI=-\overset{N}{\underset{i=1}{\sum }}\frac{L_{i}*lnL_{i}}{N}$$

where *L_i_* represents the proportion of the *i*th type of land use, and *N* is the total number of land-use types. The Cronbach alpha is 0.65 for the built-environment variables, which is considered acceptable internal reliability [47,48]. The CFI of the four variables included in the built-environment feature is 0.955, indicating a good fit.

The distribution of the built-environment features derived from different data sources is shown in Table 4. It shows that, generally, SSP has higher building density, less sky view, fewer green spaces, and lower land-use diversity than TSW.

(3) Perceived Living Environment

Individual perceived living environment was assessed by participants’ responses to questions about their general satisfaction with their living environment, perceived neighborhood pollution, and sense of community. In this study, a series of Likert scale questions ranging between 1–6 points were employed to measure four variables of perceived living environment.

First, satisfaction of the living environment was considered an indicator of people’s perceived living environment [53]. In this study, we collected participants’ overall satisfaction with their living environment as an indicator of their overall perception of the living environment.

Second, neighborhood air pollution and noise are environmental determinants of the quality of the living environment and are linked to people’s psychological stress and mental health [36,37]. As a result, participants’ perceived air pollution and noise in their neighborhood can be indicators of their perceived living environment. In the study, data concerning participants’ perceived environmental quality were collected using two questions that asked about their perception of the air pollution and noise levels in their neighborhoods (see Table 5).

In addition to perceived environmental quality, this study also explored people’s perception of the social aspects of their communities. Sense of community, including sense of community belonging, social connectivity, and social trust in the community, was linked to stress and mental health disorders including anxiety and depression [49,54]. We asked participants how much they agree with five Likert scale questions to quantify their sense of community.

The questions and coded values of the Likert scales of each variable are shown in Table 5. We used the mean value of the responses to the five questions as the result of participants’ sense of community. The internal reliability of the four variables is considered acceptable with the Cronbach alpha index of 0.64 [47,48]. The CFI value of the scale is 0.915, indicating a good fit for the perceived living environment.

(4) Reported Mental Health

We measured the self-reported mental health or mental disorders in different dimensions as mental health outcomes. Participants were asked 16 Likert scale questions in total. Each question has 1–6 points, with 1 being never and 6 being always. The mental health variables and questions are listed in Table 6.

First, we employed the World Health Organization’s Five Well-Being Indexes (WHO-5) to measure participants’ general perception of happiness and well-being [55]. The WHO-5 is a commonly used instrument for assessing clinical outcomes in controlled clinical trials and has shown to be a good measure of responsiveness/sensitivity to treatment [56]. Second, participants’ stress level was measured by self-reported frequency of distress, sleep disturbance, fatigue, and headache, which is in line with existing studies that evaluate mental health disorders [57,58]. Third, anxiety and depression level was evaluated by the well-established Patient Health Questionnaire-4 (PHQ-4), which was developed and validated by Kroenke et al. [59] and has been used widely in mental health-related research [60,61]. Lastly, recent studies found that worrying about the consequences of the COVID-19 pandemic contributed negatively to people’s mental health [62,63]. Hence, we also measured participants’ worries about life as a component of their mental health status during COVID-19. For each category of mental health-related questions, we use the mean value of the points to represent the result of the category. The Cronbach alpha index for the mental health-related variables is 0.72, which is considered satisfactory internal reliability [47,48]. The CFI for the scale of mental health is 0.987, indicating the observed variables are reliable in fitting the latent variable of mental health.

#### 2.3.2. Statistical Method

First, we conducted a non-parametric Mann–Whitney U test to examine the statistical significance of the differences in the means of the variables between the participants in SSP and TSW. Then, structural equation modeling (SEM) was used to measure the direct and indirect effects of housing and built-environment characteristics on participants’ reported mental health via perceived living environment. We employed SEM because of its advantages in modeling complex relationships between multiple causes and multiple outcomes and its wide applications in health-related studies [2,35,52]. A structural equation model is mainly composed of a measurement model and a structural model. The measurement model examines the correlation between the observed variables and the latent variables, and the structural model imputes the relationship between different latent variables. In this study, variables of housing characteristics, built-environment features, perceived living environment, and reported mental health cannot be measured directly, which are thus called latent variables. Variables that are directly measured such as housing type and perceived air pollution are observed variables. The relationships between ordinal and continuous variables in this study can be estimated by SEM based on the maximum likelihood method [64]. In the modeling effort, relationships and interactions are evaluated at the 0.1 significance level. The goodness-of-fit index (GFI), absolute goodness-of-fit (AGFI), comparative fit index (CFI), chi-square/DF, and root mean square error of approximation (RMESA) were used to assess the model fit. The SEMs were conducted using SPSS Amos.

## 3. Results

### 3.1. Descriptive Analysis

Table 7 shows the demographic characteristics of the participants in SSP and TSW. The overall age distribution in the sample recruited in SSP is higher than that in TSW, while the income level of participants in TSW is higher than that in SSP, which is in line with the census conducted in the year 2016 showing that TSW has a higher median monthly household income (25,000 HKD) than SSP (20,000 HKD). Table 7 also shows that more participants in TSW live in public rental housing and subsidized homeownership housing compared with participants in SSP, which is also consistent with the housing composition in these two selected areas (Table 1). Moreover, it can be seen from Table 7 that 9.17% of participants in SSP live in housing other than the main housing types in Table 1. The housing types of those participants include social security housing, subdivided flats, and temporary housing, indicating these participants were socioeconomically disadvantaged groups. In general, Table 7 shows that housing condition for participants in TSW is generally better than that in SSP, and SSP has more mixed housing types and contains more disadvantaged groups.

**Figure 3:**
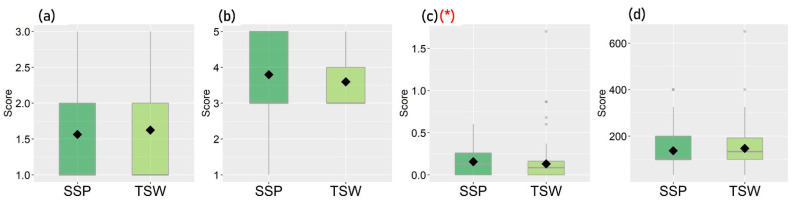
Distribution of participants’ housing characteristics (* *p* < 0.05): (**a**) Housing tenure, (**b**) Housing type, (**c**) Housing affordability stress, (**d**) Residential area per capita.

**Figure 4:**
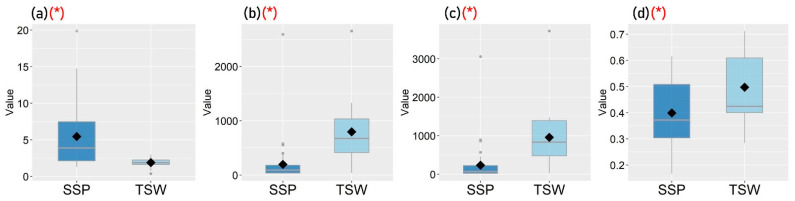
Distribution of participants’ neighborhood built-environment characteristics (* *p* < 0.05): (**a**) Building density, (**b**) Sky view factor, (**c**) Greenspaces, (**d**) Land use mix.

**Figure 5:**
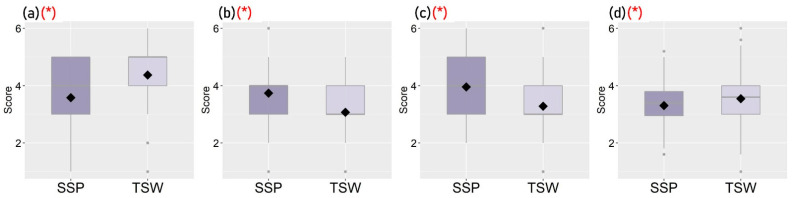
Distribution of participants’ perception of their living environment (* *p* < 0.05): (**a**) Satisfaction about living environment, (**b**) Perception about air pollution, (**c**) Perception about noise pollution, (**d**) Perception about community.

**Figure 6:**
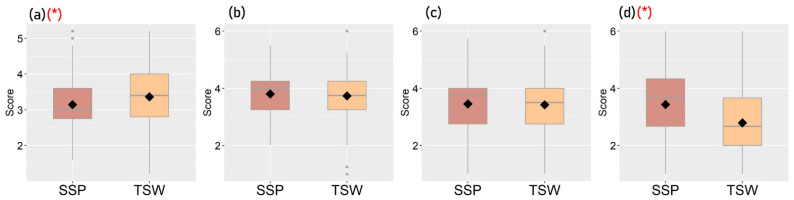
Distribution of participants’ reported mental health scores (* *p* < 0.05): (**a**) Overall happiness and well-being, (**b**) Stress, (**c**) Anxiety and depression, (**d**) Worries.

Figure 3, Figure 4, Figure 5 and Figure 6 show the distribution of each observed variable for the 221 participants in SSP and TSW. Variables with significant differences (*p* < 0.05) based on Mann–Whitney U test between groups are marked with (*) in red color. The figures show that there are significant differences in terms of housing characteristics, built-environment features, perceived living environment, and reported mental health between participants in SSP and TSW. In terms of housing characteristics, Figure 3 shows that participants living in SSP have significantly higher housing affordability stress compared with participants in TSW. It can also be seen in Figure 4 that the differences in all of the four variables of built-environment characteristics between the two communities are significant. In general, Figure 4 shows that SSP has a higher building density, less sky view and greenspaces, and lower land use diversity compared with TSW, which indicates that SSP has more crowded land use and the SSP community is not suitable for activities with diverse purposes. In fact, SSP is one of the oldest districts in Hong Kong with a high concentration of aging buildings. It is also one of the major areas involved in Hong Kong’s urban renewal programs.

Figure 5 and Figure 6 show the participants’ perceived living environment and reported mental health. Figure 5 shows that, in general, participants in TSW have higher scores on the perceived living environment. They are more satisfied with their living environment, perceive the air and noise pollution as less severe, and have a greater sense of community compared with participants in SSP. Figure 6 further shows that participants in TSW reported a higher level of overall happiness and well-being and a lower frequency of feeling worried.

While Figure 3, Figure 4, Figure 5 and Figure 6 illustrate the differences in housing characteristics, neighborhood built environment, perceived living environment, and mental health between the participants in SSP and TSW, the pathways of the effects of housing characteristics and built-environment features on mental health within each community are still unclear. Thus, statistical models or qualitative studies are needed in order to further explore the internal relationships between these variables within participants in SSP and participants in TSW.

### 3.2. Results of Structural Equation Models

We tested three competing models before setting up the current models. Model 1 hypothesizes direct relationships between housing characteristics, built environment features, and mental health, with a mediating role of perceived living environment in the relationship between housing characteristics and reported mental health. Model 2 hypothesizes the same direct relationships as Model 1 but hypothesizes a mediating role of perceived living environment in the relationship between built environment features and reported mental health. Model 3 only hypothesizes direct relationships between housing characteristics, built environment features and perceived living environment, and reported mental health. Using all data from SSP and TSW, the fitting results of Models 1, 2, and 3 as well as the proposed model are shown in Table 8, which shows that the proposed model fits the data better than the competing models.

**Figure 7:**
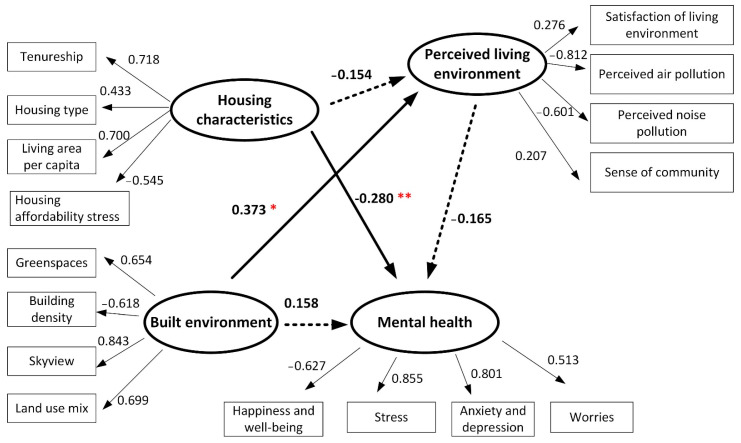
Structural equation modeling results for participants in SSP (Chi-square/DF = 1.531, GFI = 0.833, AGFI = 0.808, CFI = 0.878, RMSEA = 0.070, * *p* < 0.1, ** *p* < 0.05).

**Figure 8:**
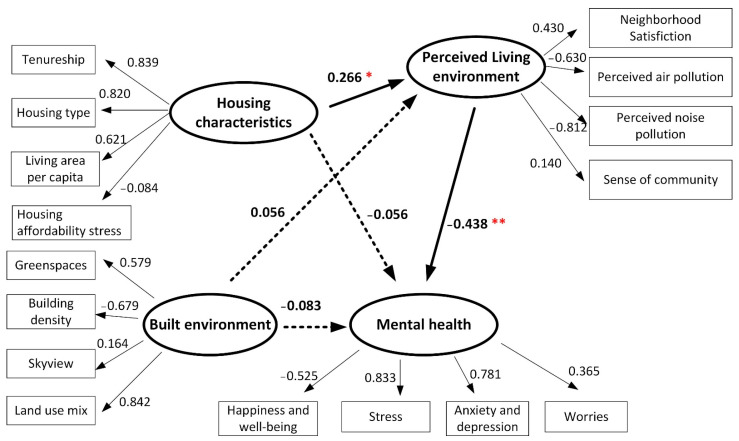
Structural equation modeling results for subjects in TSW (Chi-square/DF = 1.842, GFI = 0.836, AGFI = 0.775, CFI = 0.806, RMSEA = 0.087, * *p* < 0.1, ** *p* < 0.05).

The structural equation models shown in Figure 7 and Figure 8 examine the impacts of housing characteristics and built-environment features on mental health for participants in SSP and TSW. In the figures, a bold solid arrow with one (*p* < 0.1) or two (*p* < 0.05) red asterisks (*) indicates a significant path coefficient between the latent variables. A dashed bold arrow indicates a non-significant path coefficient between the latent variables. The solid arrows represent the standardized regression weights of the observed variables. The model fit statistics for the SSP community (Chi-square/DF = 1.531, GFI = 0.833, AGFI = 0.808, CFI = 0.878, RMSEA = 0.070) and the TSW community (Chi-square/DF = 1.842, GFI = 0.836, AGFI = 0.775, CFI = 0.806, RMSEA = 0.087) indicate a good fit of the proposed models.

For SSP participants, Figure 7 shows that housing characteristics have a direct effect on reported mental health with a path coefficient of −0.280, indicating that worse housing conditions contribute to a higher probability of a participant feeling unhappy, stressed, depressed, and worried. This significant relationship indicates that Hypothesis 1 is retained for participants living in SSP, that housing conditions have a positive and direct effect on mental health. In terms of built-environment features, Figure 7 shows that the path between the built environment and mental health is not significant, meaning that the built environment does not have a direct effect on reported mental health, which indicates that Hypothesis 2 of a direct effect of high-quality built-environment features on reported mental health is not retained for participants living in SSP. Further, the effect of the built environment on the perceived living environment has a borderline significant path coefficient of 0.373, indicating the built environment’s positive effects on the perceived living environment, including overall satisfaction with the living environment, perceived air and noise pollution, and sense of community. The path coefficient of perceived living environment upon mental health is −0.165, indicating that perceived living environment has a mild protective effect on reported mental health, while the path coefficient is not significant. It suggests that Hypothesis 3 about the mediating role of the perceived living environment between the built environment, housing characteristics, and reported mental health is not retained.

Figure 8 shows that the effect of housing characteristics, the built environment, and reported mental health for participants in TSW is different from that for participants in SSP. First, the path coefficient of housing characteristics upon mental health is not significant, indicating no direct effect of housing characteristics on reported mental health and Hypothesis 1 is not retained for participants in TSW. Although the built environment has a weak improving effect upon the perceived living environment and reported mental health, its path coefficients are not significant, indicating Hypothesis 2 is not retained either. However, Figure 8 shows a borderline significantly positive effect of housing characteristics upon perceived living environment with a path coefficient of 0.266, meaning better housing characteristics would improve people’s perceived living environment. Furthermore, perceived living environment is significantly linked with reported mental health with a path coefficient of −0.438, indicating that better perceived living environment may reduce the probability of mental disorder. The effect of housing characteristics on the perceived living environment and the effect of perceived living environment on reported mental health demonstrate the existence of a mediating role of perceived living environment between housing characteristics and mental health, and thus Hypothesis 3 is retained. The mediating effect of the perceived living environment may indicate that although housing characteristics do not have a direct effect on reported mental health for residents in TSW, improving their housing condition may still improve people’s mental health through the incremental effect on their perceived living environment.

## 4. Discussion

This study examined the effects of housing characteristics and built-environment features on mental health between participants from two communities in Hong Kong, i.e., Sham Shui Po (SSP) and Tin Shui Wai (TSW). A structural equation modeling approach was used in this study to explore both direct and indirect pathways through which housing characteristics and built-environment features influence participants’ mental health. The results show that for participants in SSP, housing characteristics have a direct and positive effect on poor mental health. For them, worse housing conditions may increase the probability that participants feel unhappy, stressed, depressed, and worried. Built environment quality has a positive effect on their perceived living environment, while this effect does not contribute to reported mental health. For participants in TSW, we found an indirect effect of housing characteristics upon reported mental health through the mediating role of perceived living environment.

Although much research has reported associations between built-environment quality and people’s mental health [29,65,66], this study found the association was somewhat weak. One possible reason is that the variations in built-environment features in these two communities (especially in SSP) were not large enough for revealing their effects on reported mental health. The weak association between built-environment features and mental health is consistent with a recent systematic review, indicating that there is very little robust public health evidence in the literature that changes to the built environment could improve mental health [67]. In existing studies, there is still a lack of consistent evidence about the association and effect of the built environment on people’s mental health. Possible reasons include different population groups studied, including the older population, children or teenagers, or specific racial/ethnic groups; different definitions of the neighborhood, ranging from participant-defined areas to Census-defined areas; different measurements of living environment features and mental health; and various analytic techniques. For example, housing conditions and the built environment may make larger differences for lower-income groups, but such differences may not be observable if the income range of the participants is not large enough to provide the income variation needed. Although the lack of robust evidence on the role of the built environment on mental health may lead to biased policymaking, addressing these issues related to inconsistent findings in environment-health research remains challenging. Our findings thus highlight the need for more studies in specific contexts with robust study designs and on the association between physical built-environment features.

This study provides evidence about the effect of housing characteristics on reported mental health in a context characterized by high population density and high housing density. Taking SSP and TSW communities as study areas, the study found that the health effects of housing characteristics may be direct. SSP residents had higher housing affordability stress, and their housing conditions were worse than residents in TSW. For them, housing characteristics (such as housing tenure and living area per person) had a direct effect on reported mental health. In comparison, TSW residents experienced less housing affordability stress and had access to a better built environment in their neighborhood. Housing characteristics did not directly produce a significant effect on reported mental health for the participants in TSW; instead, housing characteristics influenced people’s mental health through the mediating effect of perceived living environment.

Whereas previous studies have identified the associations between housing conditions and mental health, as well as the mediating role of environmental perceptions [2,68], this study further suggests that the direct or indirect relationships between housing characteristics and reported mental health may change across different communities even within the same region (i.e., Hong Kong). This phenomenon can be summarized as the spatial non-stationarity of environment–health relationships: that is, the relationships between environment and health in different geographic areas in the same region or country may be different. The main reason for the existence of spatial nonstationarity is that certain health–environment relationships are influenced by one or more spatially varying variables not included in the health–environment models [30], which may explain the differences in the effect of housing characteristics on mental health between two different communities in this study.

Furthermore, the different effects of housing characteristics on reported mental health in the two communities may reflect another type of non-stationarity, i.e., value-range non-stationarity, a version of which is referred to as the threshold effect [69,70,71]. It means that the relationships between an environmental factor and its health effects change over the observed value ranges of the environmental factor. In this study, the health effects of housing characteristics may be direct or indirect, depending on different value ranges of housing characteristics: for communities with relatively poor housing conditions (i.e., SSP in this study), the impact of housing characteristics on reported mental health may be more direct; for communities with relatively good housing conditions (i.e., TSW in this study), the effect of housing characteristics on reported mental health may be indirect. Note that the housing conditions considered in this study include housing tenure, housing affordability stress, and living space per person. This study addressed the possible spatial nonstationarity and value-range nonstationarity in the relationship between housing characteristics and mental health. Future research should thus consider the impact of nonstationarity on the analysis of environment–health relationships in order to avoid misleading findings and policy implications due to the stationarity bias [25].

Further, this study provided evidence for the relationship between housing, the built environment, and reported mental health in high-rise and dense Asian cities. Moreover, based on the comparison of the two communities, this study highlights the spatial and value-range nonstationarity in the relationship between the environment and health outcomes, which is critical for policy-making on the health impact of the environment. This study also has some limitations. First, it is cross-sectional and cannot examine the temporal associations between housing characteristics, built-environment features, and mental health and thus has limitations in revealing the causal relationships among the variables. Second, although existing research has shown a positive relationship between stress and worries about life and mental health, there is little psychometric evidence to support such measures. Third, the sample sizes of 109 and 112 in the two communities are relatively small, which might be the reason why significance levels in some of the relationships among the variables are not lower than 0.05. Future research would benefit from using a longitudinal design that enables the examination of the causal relationship between housing characteristics, built-environment features, and mental health in the two communities and analyzing the relationship between environmental factors and different mental health measures. In addition to housing tenure and type, housing affordability stress and living space per person measured in this study, future studies may also investigate the health effects of more aspects of housing characteristics such as present assets of the housing. Lastly, it will also be helpful to undertake qualitative studies on people’s views on the health effects of the living environment for different population groups based on age, gender, education, or other attributes.

## 5. Conclusions

In this study, we examined the effects of housing characteristics and the objective and perceived built environment on the reported mental health of participants from two communities with different characteristics in housing and built-environment features in Hong Kong. A structural equation modeling approach was used to explore both direct and indirect pathways through which housing characteristics and built-environment features influence participants’ reported mental health. The results showed different pathways of the effect of housing characteristics and built-environment features on reported mental health for the two communities. Our findings bring a better understanding of the effects of housing characteristics and built-environment features on mental health in high-rise and dense Asian cities.

## Figures and Tables

**Table 1:** Housing type composition in Sham Shui Po (SSP) and Tin Shui Wai (TSW).

Housing Type	SSP	TSW
Public rental housing	35.0%	58.7%
Subsidized Home Ownership Housing	5.0%	20.8%
Private permanent housing	59.0%	20.5%
Others (Temporary housing, non-residential housing, collective housing)	1.0%	0.0%
Overall	100%	100%

**Table 2:** Age distribution by gender in the study area (in mean (standard deviation)).

Gender/Community	Overall	SSP	TSW
Female	38.9 (12.4)	38.1 (11.2)	39.7 (13.6)
Male	33.9 (11.2)	37.5 (12.8)	30.7 (10.3)
Overall	36.6 (12.5)	37.8 (11.9)	35.5 (12.9)

**Table 3:** Coded values for the answers of housing characteristics.

Variables of Housing Characteristics	Participants’ Responses	Coded Value
Housing tenure	Rented	1
	Owned with a mortgage	2
	Owned without a mortgage	3
Housing affordability stress	Monthly mortgage or rent for the residential unit/monthly household income	Continuous
Type of housing	Temporary or non-residential housing	1
	Sub-divided flat	2
	Public rental housing	3
	Subsidized homeownership housing	4
	Private permanent housing	5
Living area per person	Size of residential unit/number of person living in the residential unit	Continuous

**Table 4:** Distribution of built-environment features (in mean (standard deviation)).

	SSP	TSW
Building density (count/10,000 m^2^)	5.45 (4.11)	1.90 (0.58)
Sky view factor	197.1 (366.3)	797.1 (493.8)
Greenspaces	231.5 (459.6)	959.1 (667.9)
*LUMI*	0.40 (0.12)	0.49 (0.12)

**Table 5:** Questions and coded values for the answers of the perceived living environment.

Variables	Questions	Coded Values of the Scale (1 to 6)
Overall satisfaction with the living environment	Overall, to what extent are you satisfied with your living environment?	1-Dissatisfied, 6-Very satisfied
Perceived air pollution level	To what extent do you think the air pollution in your neighborhood is serious?	1-No pollution/noise problem, 6-Very serious
Perceived noise level	To what extent do you think the noises in your neighborhood are serious?	
Sense of community	People in the community are willing to help their neighbors	1-Totally disagree, 6-Totally agree
	This is a closely related community	
	People in this community can be trusted	
	People get along well in this community	
	People in this community can deal with problems together	

**Table 6:** Mental health variables and questions.

Mental Health Variables	Questions or Response Items
Happiness and well-being: Please answer the following questions based on your feelings in the past two weeks.	I have felt cheerful and in good spirits
	I have felt calm and relaxed
	I have felt active and vigorous
	I woke up feeling fresh and rested
	My daily life has been filled with things that interest me
Stress: Over the past one year, have you been bothered by these problems?	Stressed
	Have trouble in sleeping
	Regular fatigue
	Headache
Anxiety and depression: Over the past two weeks, have you been bothered by these problems?	Feeling nervous, anxious, or on edge
	Not being able to stop or control worrying
	Feeling down, depressed, or hopeless
	Little interest or pleasure in doing things
Worries: Over the past year, how has your life been affected by COVID-19 pandemic?	Worry about unemployment
	Worry about decreasing income
	More family conflicts

**Table 7:** Demographic characteristics of participants recruited in SSP and TSW.

Variable	Description	SSP (*n* = 109)		TSW (*n* = 112)	
		*n*	Proportion	*n*	Proportion
Gender	Male	48	44.04%	52	46.43%
	Female	61	55.96%	60	53.57%
Age	18–24	17	15.60%	24	21.43%
	25–44	53	48.62%	54	48.21%
	45–64	39	35.78%	34	30.36%
Monthly household income	Less than HKD 9999	12	11.01%	9	8.04%
	HKD 10,000–19,999	37	33.94%	23	20.53%
	HKD 20,000–29,999	20	18.35%	25	22.32%
	HKD 30,000–39,000	14	12.85%	24	21.43%
	More than HKD 40,000	26	23.85%	31	27.68%
Housing type	Public rental housing	46	42.20%	61	54.46%
	Subsidized home ownership housing	4	3.67%	35	31.25%
	Private permanent housing	49	44.95%	16	14.29%
	Others (subdivided flat, etc.)	10	9.17%	0	0.00%
Overall		109	100%	112	100%

**Table 8:** Fitting results of three competing models and the proposed model.

Models	Chi-Square/DF	GFI	CFI	AGFI	RMSEA
Model 1	2.672	0.867	0.805	0.820	0.087
Model 2	2.515	0.874	0.824	0.829	0.083
Model 3	2.722	0.862	0.796	0.814	0.089
Proposed Model	2.202	0.906	0.873	0.862	0.074

## References

[1] Mair C., Roux A.D., Galea S. (2008). Are neighbourhood characteristics associated with depressive symptoms? A review of evidence. J. Epidemiol. Community Health.

[2] Xiao Y., Miao S., Sarkar C., Geng H., Lu Y. (2018). Exploring the impacts of housing condition on migrants’ mental health in nanxiang, shanghai: A structural equation modelling approach. Int. J. Environ. Res. Public Health.

[3] Galea S., Ahern J., Rudenstine S., Wallace Z., Vlahov D. (2005). Urban built environment and depression: A multilevel analysis. J. Epidemiol. Community Health.

[4] Green R.D., Kouassi M., Venkatachalam P., Daniel J. (2013). The impact of housing stressors on the mental health of a low-income African-American population. Rev. Black Political Econ..

[5] World Health Organization (WHO) Housing and Health Guidelines. http://apps.who.int/iris/bitstream/handle/10665/276001/9789241550376-eng.pdf.

[6] Baker E., Bentley R., Mason K. (2013). The mental health effects of housing tenure: Causal or compositional?. Urban Stud..

[7] Evans G.W., Palsane M.N., Lepore S.J., Martin J. (1989). Residential density and psychological health: The mediating effects of social support. J. Personal. Soc. Psychol..

[8] Singh A., Daniel L., Baker E., Bentley R. (2019). Housing disadvantage and poor mental health: A systematic review. Am. J. Prev. Med..

[9] Bentley R.J., Pevalin D., Baker E., Mason K., Reeves A., Beer A. (2016). Housing affordability, tenure and mental health in Australia and the United Kingdom: A comparative panel analysis. Hous. Stud..

[10] Park G.R., Seo B.K. (2020). Revisiting the relationship among housing tenure, affordability and mental health: Do dwelling conditions matter?. Health Soc. Care Community.

[11] Suglia S.F., Duarte C.S., Sandel M.T. (2011). Housing quality, housing instability, and maternal mental health. J. Urban Health.

[12] Sarkar C., Webster C., Gallacher J. (2014). Healthy Cities: Public Health through Urban Planning.

[13] Kearns A., Whitley E., Mason P., Bond L. (2012). ‘Living the high life’? Residential, social and psychosocial outcomes for high-rise occupants in a deprived context. Hous. Stud..

[14] Kalantari S., Shepley M. (2021). Psychological and social impacts of high-rise buildings: A review of the post-occupancy evaluation literature. Hous. Stud..

[15] Sharifi V., Amin-Esmaeili M., Hajebi A., Motevalian A., Radgoodarzi R., Hefazi M., Rahimi-Movaghar A. (2015). Twelve-month prevalence and correlates of psychiatric disorders in Iran: The Iranian Mental Health Survey, 2011. Arch. Iran. Med..

[16] Romans S., Cohen M., Forte T. (2011). Rates of depression and anxiety in urban and rural Canada. Soc. Psychiatry Psychiatr. Epidemiol..

[17] Wiens K., Williams J.V., Lavorato D.H., Bulloch A.G., Patten S.B. (2017). The prevalence of major depressive episodes is higher in urban regions of Canada. Can. J. Psychiatry.

[18] Zijlema W.L., Klijs B., Stolk R.P., Rosmalen J.G. (2015). (Un) healthy in the city: Respiratory, cardiometabolic and mental health associated with urbanity. PLoS ONE.

[19] Chen J., Chen S., Landry P.F., Davis D.S. (2014). How dynamics of urbanization affect physical and mental health in urban China. China Q..

[20] Wang R., Xue D., Liu Y., Chen H., Qiu Y. (2018). The relationship between urbanization and depression in China: The mediating role of neighborhood social capital. Int. J. Equity Health.

[21] Okulicz-Kozaryn A. (2015). Happiness and Place: Why Life is Better Outside of the City.

[22] Blay S.L., Schulz A.J., Mentz G. (2015). The relationship of built environment to health-related behaviors and health outcomes in elderly community residents in a middle income country. J. Public Health Res..

[23] Beyer K.M., Kaltenbach A., Szabo A., Bogar S., Nieto F.J., Malecki K.M. (2014). Exposure to neighborhood green space and mental health: Evidence from the survey of the health of Wisconsin. Int. J. Environ. Res. Public Health.

[24] Wood L., Hooper P., Foster S., Bull F. (2017). Public green spaces and positive mental health–investigating the relationship between access, quantity and types of parks and mental wellbeing. Health Place.

[25] Dadvand P., Bartoll X., Basagaña X., Dalmau-Bueno A., Martinez D., Ambros A., Cirach M., Triguero-Mas M., Gascon M., Borrell C. (2016). Green spaces and general health: Roles of mental health status, social support, and physical activity. Environ. Int..

[26] Simone C., Carolin L., Max S., Reinhold K. (2013). Associations between community characteristics and psychiatric admissions in an urban area. Soc. Psychiatry Psychiatr. Epidemiol..

[27] Khan N.Y., Ghafoor N., Iftikhar R., Malik M. (2012). Urban annoyances and mental health in the city of Lahore, Pakistan. J. Urban Aff..

[28] Miles R., Coutts C., Mohamadi A. (2012). Neighborhood urban form, social environment, and depression. J. Urban Health.

[29] Melis G., Gelormino E., Marra G., Ferracin E., Costa G. (2015). The effects of the urban built environment on mental health: A cohort study in a large northern Italian city. Int. J. Environ. Res. Public Health.

[30] Kwan M.-P. (2021). The stationarity bias in research on the environmental determinants of health. Health Place.

[31] Ng M.K., Yeung T.C., Kwan M.P., Tieben H., Lau T.Y.T., Zhu J., Xu Y. (2021). Place qualities, sense of place and subjective well-being: A study of two typical urban neighbourhoods in Hong Kong. Cities Health.

[32] Wang J., Long R., Chen H., Li Q. (2019). Measuring the psychological security of urban residents: Construction and validation of a new scale. Front. Psychol..

[33] Zhang L., Wu L. (2021). Effects of environmental quality perception on depression: Subjective social class as a mediator. Int. J. Environ. Res. Public Health.

[34] Pedersen E., Waye K.P. (2007). Wind turbine noise, annoyance and self-reported health and well-being in different living environments. Occup. Environ. Med..

[35] Kou L., Tao Y., Kwan M.-P., Chai Y. (2020). Understanding the relationships among individual-based momentary measured noise, perceived noise, and psychological stress: A geographic ecological momentary assessment (GEMA) approach. Health Place.

[36] Dzhambov A.M., Markevych I., Tilov B., Arabadzhiev Z., Stoyanov D., Gatseva P., Dimitrova D.D. (2018). Pathways linking residential noise and air pollution to mental ill-health in young adults. Environ. Res..

[37] Tao Y., Kou L., Chai Y., Kwan M.-P. (2021). Associations of co-exposures to air pollution and noise with psychological stress in space and time: A case study in Beijing, China. Environ. Res..

[38] Chen C., Luo W., Li H., Zhang D., Kang N., Yang X., Xia Y. (2020). Impact of perception of green space for health promotion on willingness to use parks and actual use among young urban residents. Int. J. Environ. Res. Public Health.

[39] Kothencz G., Kolcsár R., Cabrera-Barona P., Szilassi P. (2017). Urban green space perception and its contribution to well-being. Int. J. Environ. Res. Public Health.

[40] Heerman W.J., Mitchell S.J., Thompson J., Martin N.C., Sommer E.C., Van Bakergem M., Taylor J.L., Buchowski M.S., Barkin S.L. (2016). Parental perception of built environment characteristics and built environment use among Latino families: A cross-sectional study. BMC Public Health.

[41] McGinn A.P., Evenson K.R., Herring A.H., Huston S.L., Rodriguez D.A. (2007). Exploring associations between physical activity and perceived and objective measures of the built environment. J. Urban Health.

[42] Lee G.K., Chan E.H. (2008). Factors affecting urban renewal in high-density city: Case study of Hong Kong. J. Urban Plan. Dev..

[43] He Y., Talamini G., Jiang L. (2021). Does urban renewal impact social interaction in public open space? Evidence from Sham Shui Po, Hong Kong. Urbanie Urbanus.

[44] Mason K.E., Baker E., Blakely T., Bentley R.J. (2013). Housing affordability and mental health: Does the relationship differ for renters and home purchasers?. Soc. Sci. Med..

[45] Bentley R., Baker E., Mason K., Subramanian S.V., Kavanagh A.M. (2011). Association between housing affordability and mental health: A longitudinal analysis of a nationally representative household survey in Australia. Am. J. Epidemiol..

[46] Gou Z., Xie X., Lu Y., Khoshbakht M. (2018). Quality of Life (QoL) survey in Hong Kong: Understanding the importance of housing environment and needs of residents from different housing sectors. Int. J. Environ. Res. Public Health.

[47] Churchill G.A., Suprenant C. (1979). A paradigm for developing better measures for marketing of consumer satisfaction. J. Mark..

[48] Taber K.S. (2018). The use of Cronbach’s alpha when developing and reporting research instruments in science education. Res. Sci. Educ..

[49] French S., Wood L., Foster S.A., Giles-Corti B., Frank L., Learnihan V. (2014). Sense of community and its association with the neighborhood built environment. Environ. Behav..

[50] Wang P., Goggins W.B., Zhang X., Ren C., Lau K.K.L. (2020). Association of urban built environment and socioeconomic factors with suicide mortality in high-density cities: A case study of Hong Kong. Sci. Total Environ..

[51] Yang J., Wong M.S., Menenti M., Nichol J. (2015). Modeling the effective emissivity of the urban canopy using sky view factor. ISPRS J. Photogramm. Remote Sens..

[52] Guo Y., Liu Y., Lu S., Chan O.F., Chui C.H.K., Lum T.Y.S. (2021). Objective and perceived built environment, sense of community, and mental wellbeing in older adults in Hong Kong: A multilevel structural equation study. Landsc. Urban Plan..

[53] Chen J., Chen S. (2015). Mental health effects of perceived living environment and neighborhood safety in urbanizing China. Habitat Int..

[54] Rugel E.J., Carpiano R.M., Henderson S.B., & Brauer M. (2019). Exposure to natural space, sense of community belonging, and adverse mental health outcomes across an urban region. Environ. Res..

[55] World Health Organization (WHO) (1998). Wellbeing Measures in Primary Health Care/the DepCare Project: Report on a WHO Meeting: Stockholm, Sweden, 12–13 February 1998.

[56] Topp C.W., Østergaard S.D., Søndergaard S., Bech P. (2015). The WHO-5 Well-Being Index: A systematic review of the literature. Psychother. Psychosom..

[57] Ma J., Li C., Kwan M.P., Chai Y. (2018). A multilevel analysis of perceived noise pollution, geographic contexts and mental health in Beijing. Int. J. Environ. Res. Public Health.

[58] Tao Y., Yang J., Chai Y. (2020). The anatomy of health-supportive neighborhoods: A multilevel analysis of built environment, perceived disorder, social interaction and mental health in Beijing. Int. J. Environ. Res. Public Health.

[59] Kroenke K., Spitzer R.L., Williams J.B., Löwe B. (2009). An ultra-brief screening scale for anxiety and depression: The PHQ–4. Psychosomatics.

[60] Pouso S., Borja Á., Fleming L.E., Gómez-Baggethun E., White M.P., Uyarra M.C. (2021). Contact with blue-green spaces during the COVID-19 pandemic lockdown beneficial for mental health. Sci. Total Environ..

[61] Jalloh M.F., Li W., Bunnell R.E., Ethier K.A., O’Leary A., Hageman K.M., Redd J.T. (2018). Impact of Ebola experiences and risk perceptions on mental health in Sierra Leone, July 2015. BMJ Glob. Health.

[62] Blix I., Birkeland M.S., Thoresen S. (2021). Worry and mental health in the Covid-19 pandemic: Vulnerability factors in the general Norwegian population. BMC Public Health.

[63] Kämpfen F., Kohler I.V., Ciancio A., Bruine de Bruin W., Maurer J., Kohler H.P. (2020). Predictors of mental health during the Covid-19 pandemic in the US: Role of economic concerns, health worries and social distancing. PLoS ONE.

[64] Skrondal A., Rabe-Hesketh S. (2005). Structural equation modeling: Categorical variables. Encycl. Stat. Behav. Sci..

[65] Weich S., Blanchard M., Prince M., Burton E., Erens B., Sproston K. (2002). Mental health and the built environment: Cross–sectional survey of individual and contextual risk factors for depression. Br. J. Psychiatry.

[66] Guzman V., Garrido-Cumbrera M., Braçe O., Hewlett D., Foley R. (2021). Associations of the natural and built environment with mental health and wellbeing during COVID-19: Irish perspectives from the GreenCOVID study. Lancet Glob. Health.

[67] Moore T., Kesten J., López-López J.A., Ijaz S., McAleenan A., Richards A., Gray S., Savović J., Audrey S. (2018). The effects of changes to the built environment on the mental health and well-being of adults: Systematic review. Health Place.

[68] Domènech-Abella J., Switsers L., Mundó J., Dierckx E., Dury S., De Donder L. (2021). The association between perceived social and physical environment and mental health among older adults: Mediating effects of loneliness. Aging Ment. Health.

[69] Tran B.-L., Chang C.-C., Hsu C.-S., Chen C.-C., Tseng W.-C., Hsu S.-H. (2019). Threshold Effects of PM2. 5 exposure on particle-related mortality in China. Int. J. Environ. Res. Public Health.

[70] Wu X., Tao T., Cao J., Fan Y., Ramaswami A. (2019). Examining threshold effects of built environment elements on travel-related carbon-dioxide emissions. Transp. Res. Part D Transp. Environ..

[71] Zhang L., Zhou S., Kwan M.-P., Chen F., Dai Y. (2020). The threshold effects of bus micro-environmental exposures on passengers’ momentary mood. Transp. Res. Part D Transp. Environ..

